# Supplementary material for: Development and utilization of Treponema pallidum expressing green fluorescent protein to study spirochete-host interactions and antibody-mediated clearance: expanding the toolbox for syphilis research
Source: mBio. 2024 Nov 29;16(1):e03253-24. doi: 10.1128/mbio.03253-24 (PMC11708019; doi:10.1128/mbio.03253-24)
Supplement: Supplemental Material — Tables S1 and S2; Figures S1-S5; supplemental video legends. [file mbio.03253-24-s0001.pdf]

Supplementary Material

Development and utilization of *Treponema pallidum* expressing green fluorescent protein to study spirochete-host interactions and antibody-mediated clearance: expanding the toolbox for syphilis research.

Kristina N. Delgado[1], Crystal F. Vicente[2], Christopher M. Hennelly[3], Farhang Aghakhanian[3], Jonathan B. Parr[3, 4], Kevin P. Claffey[5], Justin D. Radolf[1, 6-9], Kelly L. Hawley[1,2,7,9, #], and Melissa J. Caimano[1,2,6,9,*,#]

Departments of [1]Medicine, [2]Pediatrics, [5]Cell Biology, and [6]Molecular Biology and Biophysics, [7]Immunology and [8]Genetics and Genome Sciences, University of Connecticut Health, Farmington, CT, USA
[3]Institute for Global Health and Infectious Diseases and [4]Division of Infectious Diseases, Department of Medicine, University of North Carolina at Chapel Hill, Chapel Hill, North Carolina, USA
[9]Connecticut Children's Research Institute, Connecticut Children's, Hartford, Connecticut, USA

Table S1. Oligonucleotide primers used in this study.

Name	Sequence (5'-3')	Purpose
tprA-FW	CGACTCTAGAGGATCCTCCACAGCACAGTATCTTTGTTTTG	*tprA* plus flanking sequence
tprA-RV	CGGTACCCGGGGATCCTCTGTAGATGATGTCCTTGCG	*tprA* plus flanking sequence
tprAvec-F	CCCAGGGAGACGGCGACA	Insertion of tprA into pUC19
tprAvec-R	AGTTTAGTACAACGATGTCATGTGT	Insertion of tprA into pUC19
47k-kan-F	TCGTTGTACTAAACTAGCGGATCCTCCCAAAAAGAG	Kanamycin-resistance cassette used for allelic replacement of *tprA*
47k-kan-R	CGCCGTCTCCCTGGGTCAGAAAAACTCATCGAGCATC	Kanamycin-resistance cassette used for allelic replacement of *tprA*
gfpvec-F	TCTTCCCACCCACGAATATCTAATG	Insertion of extra superfolder-*gfp*
gfpvec-R	AGAGTCACTTTCTGGGGAGGC	Insertion of extra superfolder-*gfp*
gfpIns-F	CCAGAAAGTGACTCTCTACTTGTAAAGCTCATCCATCCCG	Insertion of extra superfolder-*gfp*
gfpIns-R	TCGTGGGTGGGAAGAGGACGTGTAGTCTGCACCG	Insertion of extra superfolder-*gfp*
tprB3'-F	CATGCTCCAAACTTATGTAGGGAC	Confirm *gfp-kan* insertion in *TPA*
tprAups-F	TCGCAGCAGCAACAAGTAACCC	Confirm *gfp-kan* insertion in *TPA*
tprA-KO FW	CGTATGCTTTTACCCGCTGT	Confirm *gfp-kan* insertion in *TPA*
gfp3'-F	ACGAGTTTGTGACGGCGGCGGGGATT	Confirm *gfp-kan* insertion in *TPA*
gfpPCR-F	TCCCCCTCAAACTTCACCTCC	Confirm *gfp-kan* insertion in *TPA*
gfpPCR-R	ATTCTTGGGCACAAGCTTGAG	Confirm *gfp-kan* insertion in *TPA*
kanPCR-F	ATGAGCCATATTCAACGGGAGACG	Confirm *gfp-kan* insertion in *TPA*
kan3'-F	GCCATCCTATGGAACTGCCTCGGTGAA	Confirm *gfp-kan* insertion in *TPA*
kan3'-R	GATGCTCGATGAGTTTTTCTGACCCAGGGAGACGGCG	Confirm *gfp-kan* insertion in *TPA*
tprAdwns-R	GGTAATGGGCTCTGGGGTAT	Confirm *gfp-kan* insertion in *TPA*
Tp polA-F	CAGGATCCGGCATATGTCC	qPCR
Tp polA-R	AAGTGTGAGCGTCTCATCATTCC	qPCR
polA-probe	(6FAM) CTGTCATGCACCAGCTTCGACGTCTT (BHQ1)	qPCR

Supplemental Table 2. Nucleotide polymorphisms detected in GFP[+] *TPA* Nichols strain by whole-genome sequencing

Coord[a]	Locus[a]	Product[a]	Type[b]	Nucleotide difference[c]	Passage[d]	Amino acid effect	Nichols parent[e]
7179	TP_0006	Pseudogene	SNP	T → C	all	N/A	Y
7543	TPANIC_ RS00035	Pseudogene	1-bp del	$TG_5 \rightarrow TG_4$	all	N/A	
34076	TP_0027	HlyC/CorC family transporter	1-bp ins	$GC_9 \rightarrow GC_{10}$	all	Frameshift	
49360	**TP_0040**	Methyl-accepting chemotaxis protein (Mcp1)	2-bp ins	$CG_{10} \rightarrow CG_{12}$	all	Frameshift at C-terminus	
59894	**TP_0051**	Peptide chain release factor 1	SNP	T → C	all	Ser → Pro	Y
72679	TP_0067	Hypothetical protein	2-bp del	$CG_9 \rightarrow CG_7$	all	Frameshift at N-terminus	
148350	TP_0124	Hypothetical protein	1-bp ins	$AC_9 \rightarrow AC_{10}$	all	C-terminal extension	
149360	noncoding	Noncoding	SNP	C→T	all	N/A	Y
150148	**TP_0127**	DUF2715 domain-containing protein	1-bp del	$TG_{10} \rightarrow TG_9$	all	Frameshift	
159101	TP_0136	Hypothetical protein	SNP	G → A	IV P12	Gly → Ser	Y
231090	TP_0225	Hypothetical protein	1-bp del	$TG_5 \rightarrow TG_4$	all	Frameshift at C-terminus	Y
335831	noncoding	Noncoding	2-bp ins	$AC_{10} \rightarrow AC_{12}$	Rb1	N/A	
373207	TPANIC_ RS01700	DUF2715 domain-containing protein	1-bp del	$CG_{13} \rightarrow CG_{12}$	all	Frameshift	
386978	TP_0363	Chemotaxis protein (CheA)	SNP	A → G	all	Lys → Glu	Y
409104	noncoding	Noncoding	1-bp ins	$AC_{12} \rightarrow AC_{13}$	IV P6, Rb1, Rb2	N/A	Y
671435	**TP_0618**	DUF2715 domain-containing protein	1-bp del	$AC_9 \rightarrow AC_8$	all	Frameshift at C-terminus	
674514	N	noncoding	1-bp ins	$AC_8 \rightarrow AC_9$	IV P12	N/A	
741934	TP_0674	Hypothetical protein	SNP	C → T	All	Ser → Leu	Y
747420	TP_0681	Alanine racemase	SNP	C → T	all	Ala → Thr	
789604	TP_0720	Flagellar motor switch protein FliN	SNP	G → A	all	Ala → Val	Y
809311	TP_0742	GTPase ObgE	SNP	A → G	all	silent	Y
810792	TP_0746	Pyruvate, phosphate dikinase	SNP	G → A	all	Ser → Leu	
874544	TP_0804	Sugar ABC transporter ATP-binding protein	SNP	G → A	all	Asp → Asn	
928188	TP_0854	Hypothetical protein	SNP	G → A	all	Thr → Met	Y
929715	TP_0854	Hypothetical protein	SNP	C → T	all	Gly → Asp	Y
938003	TP_0859	Hypothetical protein	1-bp del	$CG_{10} \rightarrow CG_9$	all	Frameshift	
975816	noncoding	Noncoding	67-bp del	deletion	all	N/A	Y
1023862	TP_0939	Pyruvate:ferredoxin (flavodoxin) oxidoreductase	SNP	G → A	all	silent	Y
1125669	noncoding	Noncoding	2-bp-del	$CG_{11} \rightarrow CG_9$	all	N/A	Y

Bold, variant observed by Edmondson *et al*. (33)

a. Coordinate, locus ID and product description based on *TPA* Nichols genome (CP004010.2)
b. SNP, single nucleotide polymorphism.
c. Subscripts indicate the number of homopolymeric bases.
d. All, same polymorphism found in all passages examined. IV P#, *in vitro* passage number.
e. *TPA* Farmington Nichols parent used to generate the GFP[+] strain used in these studies.

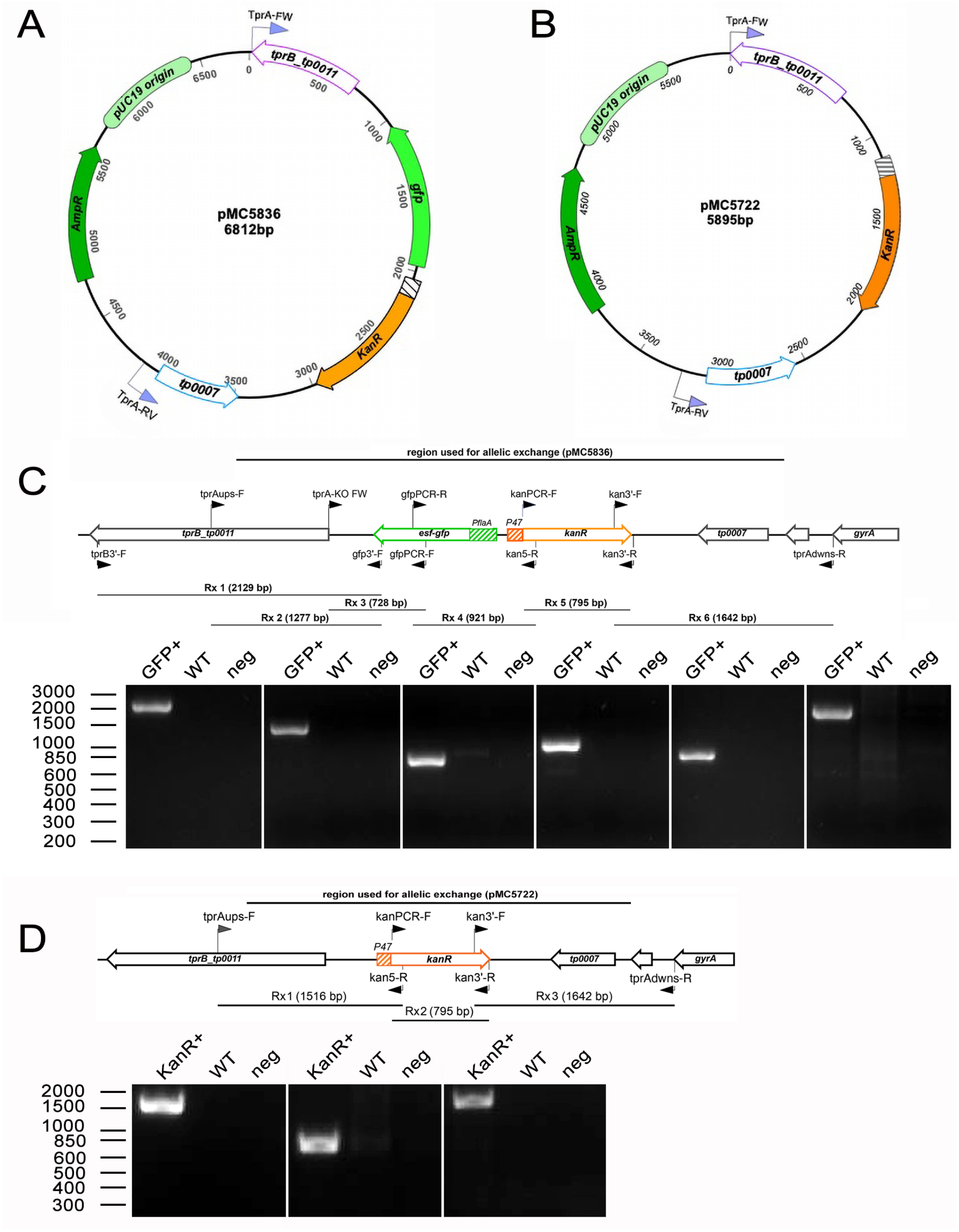

Fig S1. Replacement of *tprA* with extra-superfolder GFP and/or a kanamycin resistance gene. Plasmids used to replace *tprA* in the *TPA* Nichols chromosome with a constitutively expressed codon-optimized extra-superfolder green fluorescent protein (*gfp*) transgene under the control of the *flaA1* promoter and a kanamycin-resistance gene (*kanR*) from *Proteus mirabilis* under the control of the *tpp47* promoter (pMC5836) (**A**) or the *kanR* cassette alone (**B**). Schematics showing the chromosomal regions used to insert the *gfp-kanR* (**C**) and *kanR* (**D**) cassettes. Arrows are used to indicate locations of primers (Table S1) used to confirm insertion. The expected size in base pairs (bp) for each amplicon is indicated above the corresponding line. Agarose gel images showing the corresponding PCR amplicons obtained using genomic DNA from GFP$^+$ (**C**), *kanR* (**D**) *TPA* Nichols strains are shown below. Genomic DNA from WT *TPA* was used as a negative (no insert) control. Neg, No DNA control. DNA ladder (bp) is shown on the left of gels in **C** and **D**.

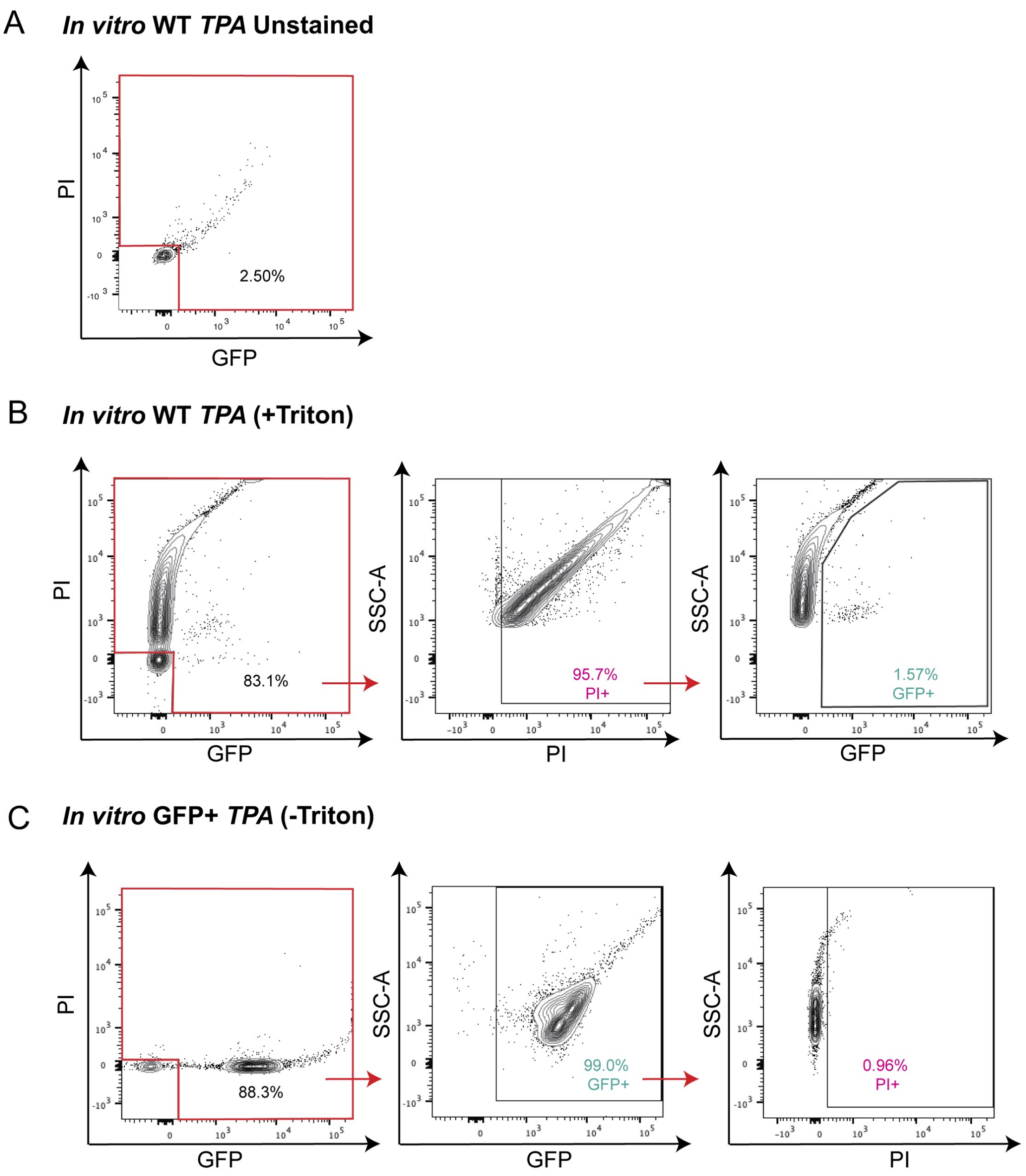

Fig S2. Gating strategies used for flow cytometric analysis of WT and GFP$^+$ *TPA*. (**A**) Flow cytometry panels for unstained WT *TPA* used to define and exclude non-spirochetal (*i.e.*, double-negative) events. (**B**) Flow cytometry panels for detergent-treated (+ Triton), *in vitro*-cultivated WT *TPA* stained with PI used to define the spirochete population. (**C**) Flow cytometry panels for, *in vitro*-cultivated GFP$^+$ *TPA* stained with PI in the absence of detergent (- Triton) used to define the GFP$^+$ population and confirm exclusion of PI by intact treponemes. Results are representative of three independent experiments.

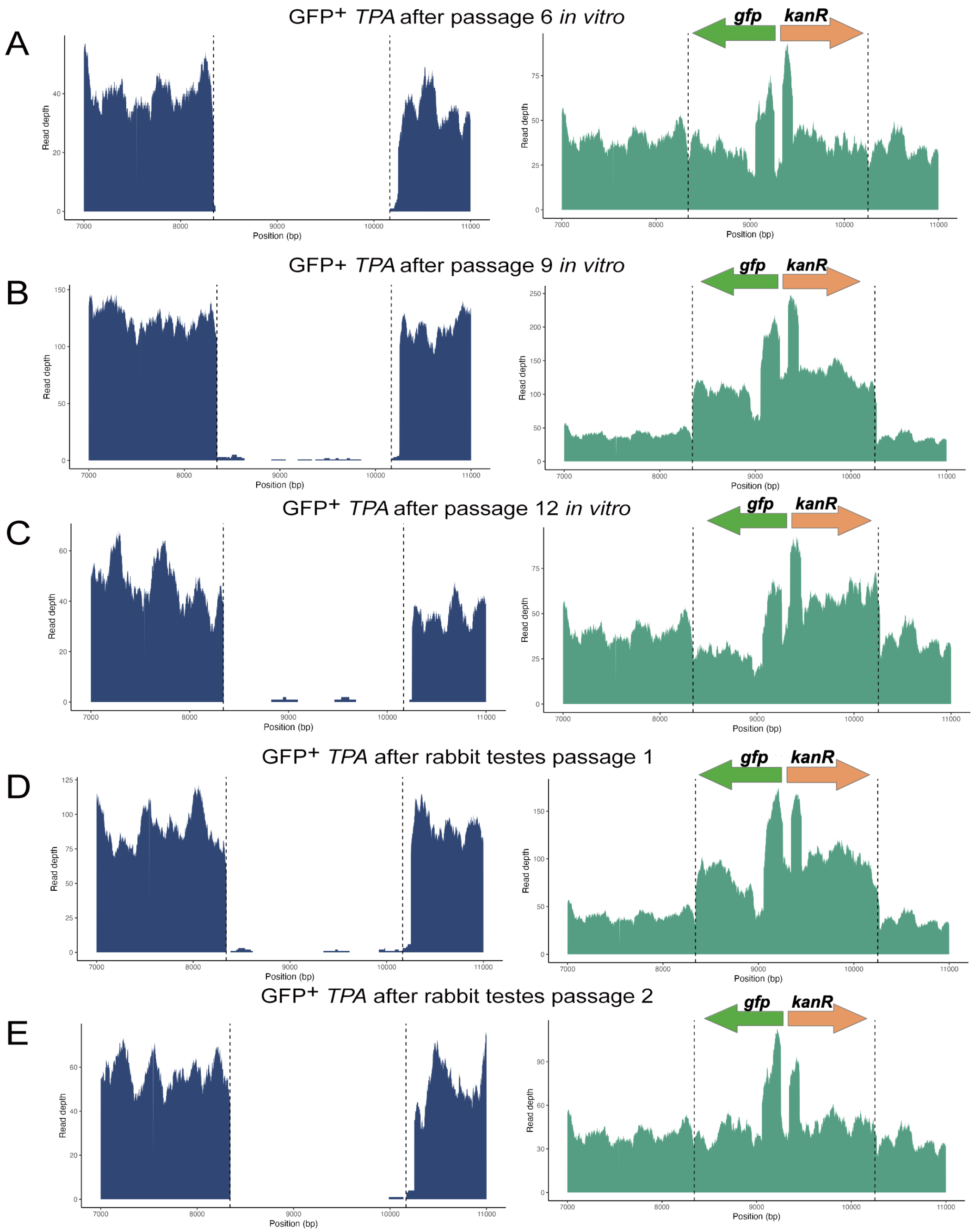

Fig S3. Whole-genome sequencing to confirm replacement of *tprA* with *gfp-kanR* in GFP⁺ *TPA*. Genome sequencing confirms the replacement of *tprA* with the *gfp-kanR* cassette in GFP⁺ *TPA*. Assembled reads for *tprA* and flanking regions from GFP⁺ *TPA* after 6, 9 and 12 passages *in vitro* (**A-C**) and serial passages in rabbit testes (**D**, **E**) mapped against the *TPA* Nichols reference genome (left panels) and modified genome containing the *gfp-kanR* transgenes in place of *trpA* (right panels). The gaps in coverage in A-E left panels demonstrate complete replacement of the *trpA* coding sequence in GFP⁺ *TPA*.

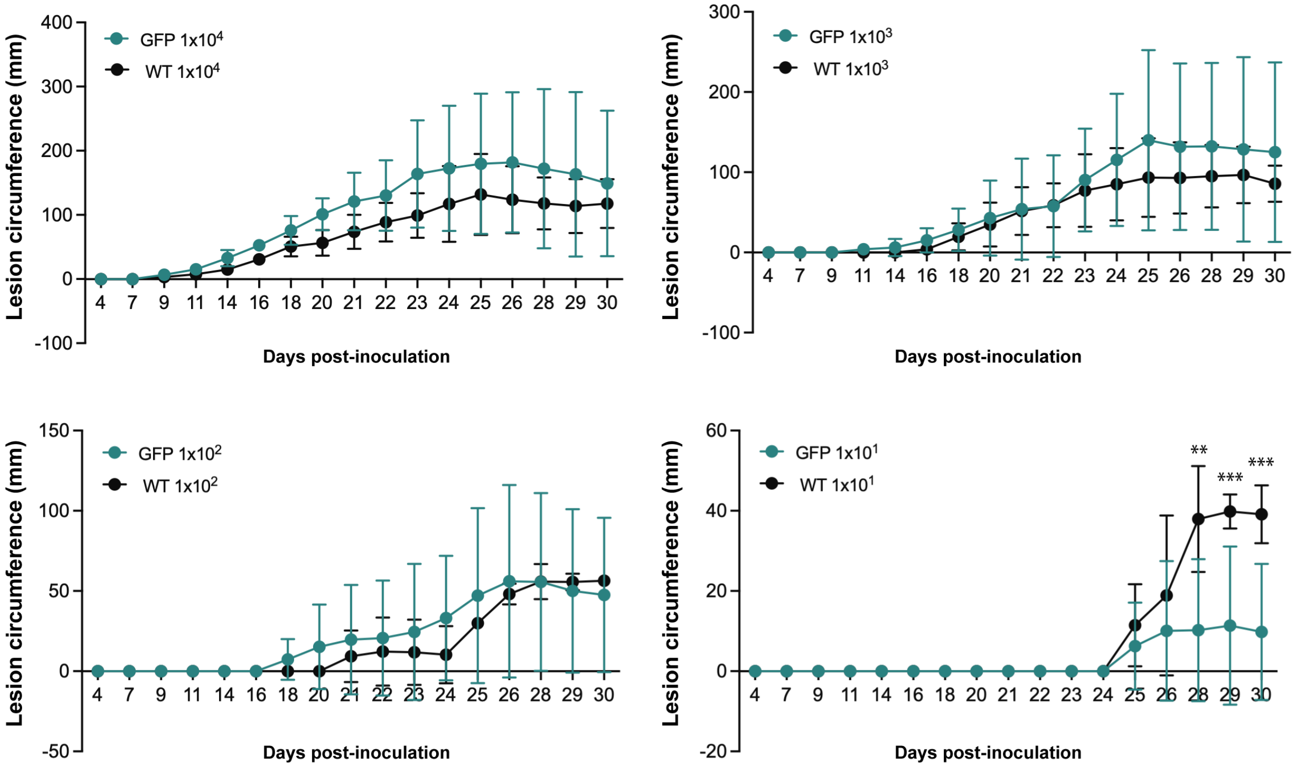

Fig S4. Rabbit intradermal inoculations with GFP^+ *TPA* mirrors lesion development of WT *TPA*. Lesion circumferences measured in mm and averaged from rabbits (n = 3) inoculated intradermally with graded doses ($1x10^4$ – $1x10^1$) of GFP^+ and WT *TPA.* Lesions were measured beginning day 7 p.i. until sacrifice (day 30 p.i.). Values represent the mean ± standard deviation for three biological replicates per condition. **, $p \leq 0.01$; or ***$p \leq 0.001$.

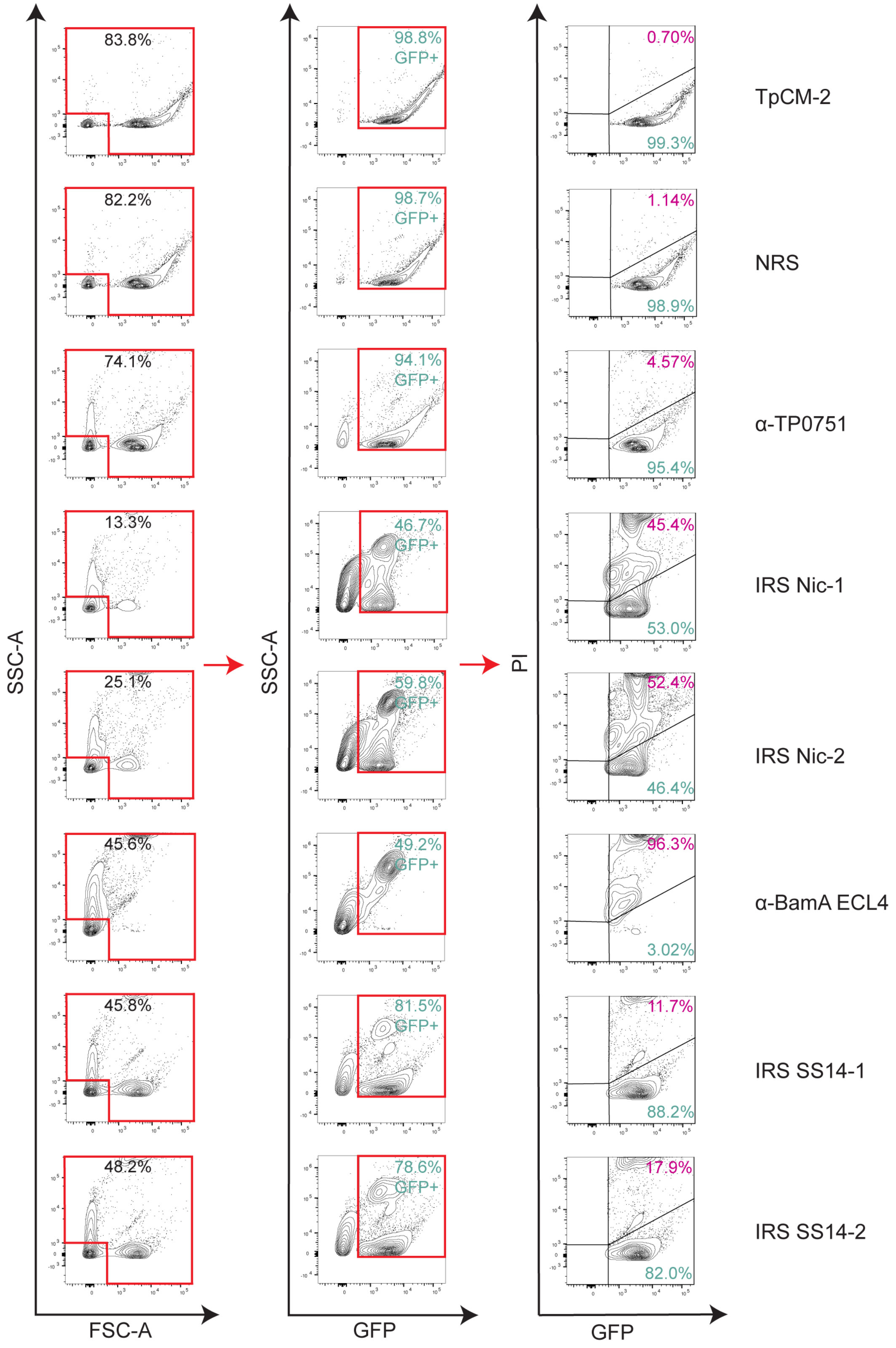

Fig S5. Gating strategy used to assess OM disruption of *in vitro*-cultivated GFP$^+$ *TPA*. Flow cytometric panels used to exclude non-spirochetal (*i.e.*, double negative) events and then assessing the percentage of PI$^+$ organisms within the GFP$^+$ population for each serum. Results are representative of three independent experiments.

Video S1. Epifluorescence video showing motility of *in vitro*-cultivated GFP$^+$ *TPA* Nichols strain.

Video S2. Epifluorescence video showing motility of *kanR TPA* Nichols strain.

Video S3. Z-stack of individual 1 μm optical sections showing surface localization of *in vitro*-cultivated GFP$^+$ *TPA* Nichols strain co-cultured with Sf1Ep rabbit epithelial cells.

Video S4. Epifluorescence video showing motility of GFP$^+$ *TPA* Nichols strain harvested from rabbit testes.

Video S5. Epifluorescence video showing motility of WT *TPA* Nichols strain harvested from rabbit testes.

Video S6. Z-stack of individual 1 μm optical sections showing surface localization of GFP$^+$ *TPA* Nichols strain harvested from rabbit testes.
